# Neurons Self-Organize Around Salivary Epithelial Cells in Novel Co-Culture Model

**DOI:** 10.15436/2471-0598.16.013

**Published:** 2016-05-27

**Authors:** Salah Sommakia, Olga J. Baker

**Affiliations:** School of Dentistry, the University of Utah, Salt Lake City, UT, USA

**Keywords:** Salivary gland, Neuron, Co-culture

## Abstract

Salivary gland bioengineering requires understanding the interaction between salivary epithelium and surrounding tissues. An important component of salivary glands is the presence of neurons. No previous studies have investigated how neurons and salivary epithelial cells interact in an *in vitro* co-culture model. In this study, we describe the self-organization of neurons around salivary epithelial cells in co-culture, in a similar fashion to what occurs in native tissue. We cultured primary mouse cortical neurons (m-CN) with a salivary epithelial cell line (Par-C10) on growth factor-reduced Matrigel (GFR-MG) for 4 days. After this time, co-cultures were compared with native salivary glands using confocal microscopy. Our findings indicate that m-CN were able to self-organize basolaterally to salivary epithelial cell clusters in a similar manner to what occurs in native tissue. These results indicate that this model can be developed as a potential platform for studying neuron-salivary epithelial cell interactions for bioengineering purposes.

## Introduction

Hyposalivation results in poor oral health and negatively affects patients’ quality of life^[1]^. The most prominent cause of this condition is primary Sjögren’s Syndrome (SS), an autoimmune disease that affects salivary and lacrimal glands, but it can also result from a wide variety of oral and systemic diseases, such as diabetes, depression, fibromyalgia, and HIV/AIDS, in addition to iatrogenic causes such as radiation therapy, chemotherapy, and drugs^[2]^. Current treatments for hyposalivation include: a) saliva substitutes which only provide temporary relief, b) systemic therapies such as cholinergic secretory agonists which are hampered by side effects and contraindications, and c) other experimental therapies (example: gene therapy, electrostimulation) that are either not sufficiently effective or require residual secretory levels^[3]^.

An additional treatment option is the implantation of a bioengineered artificial salivary gland. Current research investigates a variety of growth substrates but is limited to salivary gland primary cells and cell lines, while other cellular components necessary for proper salivary gland function are lacking^[4]^. For example, salivary gland innervation is essential for proper secretory function, given that saliva secretion is initiated by acetylcholine release from post-ganglionic neurons to activate muscarinic type 3 receptors in acinar cells, leading to fluid secretion^[5]^. Understanding neuron-salivary epithelial cell interactions is an important step for the development of a viable artificial salivary gland. One valuable approach to study cell-cell interactions is the use of co-cultures^[6]^. Neurons have been shown to thrive in co-culture with endothelial cells^[7]^ and skeletal muscle cells^[8]^, and while the role of parasympathetic innervation of salivary glands has been investigated in embryonic submandibular organ explant cultures^[9]^, no studies to date have investigated co-cultures of neurons and salivary epithelial single cells. In order to develop an *in vitro* model to study functional interactions between neurons and salivary epithelial cells, the following conditions have to be demonstrated: 1) ability of cells to thrive when cultured together, 2) that neurons do not cause salivary epithelial disruption, or vice versa, and 3) ability of neurons to localize on the basolateral surface of salivary epithelial cell clusters.

The main goal of this study was to verify the existence of precursor conditions under which co-cultures of neuron-salivary epithelial cells physically organize in a similar fashion to native tissue. Specifically, our *in vitro* model is a co-culture of primary mouse cortical neurons (m-CN) and a salivary epithelial cell line (Par-C10) cells. The Par-C10 immortalized cell line was derived by Quissel *et al.* In 1998 from rat parotid gland acinar cells by transformation with simian virus 40, and at high passage numbers, i.e. 40 - 60, exhibits many characteristics of freshly isolated acinar cells^[10,11]^. Par-C10 cells have been widely used as a model for studying salivary gland epithelial integrity. Particularly, they form a polarized epithelium when grown on permeable supports and on Matrigel and are able to respond to salivary secretory agonists^[4,12–15]^. Primary neurons have been used extensively to create *in vitro* models of the nervous system^[16]^. Our findings indicate that m-CN were able to self-organize basolaterally to salivary epithelial cell clusters in a similar manner to what occurs in native tissue. We propose that this model can be developed as a potential platform to study neuron-salivary epithelial cell interactions *in vitro* for the purposes salivary gland bioengineering applications.

## Materials and Methods

### Experimental animals

Submandibular gland extractions were performed on C57BL/6 mice for tissue analysis. The animals were anesthetized via intraperitoneal (IP) injection with 80 to 100 mg/kg ketamine and 5 mg/kg xylazine, and euthanized by abdominal exsanguination. SMG were then removed and snap-frozen and sliced into 5 µm-thick sections. All animal usage, anesthesia, and surgery were conducted with the approval of the University of Utah Institutional Animal Care and Use Committee (IACUC), in accordance with their strict guidelines (IACUC protocol number 14-06012, June 26^th^ 2014).

### Substrate preparation

The growth substrate was prepared by mixing growth factor reduced Matrigel (GFR-MG) (Becton Dickinson Labware, Franklin Lakes, NJ) at a 3:1 ratio with DMEM/Ham’s F12 (1:1) serum-free medium (Hyclone, Logan, UT). Ninety microliters of the diluted GFR-MG were placed in each well of a sixteen-well chamber mounted on #1.5 German borosilicate coverglass (Grace Bio-Labs, Bend, OR), and allowed to solidify for 1 h in a 37°C incubator with 95% air and 5% CO_2_.

### Cell plating

Par-C10 cells (passage 50–60) were prepared from a confluent flask. m-CN (Life Technologies, Carlsbad, CA) were prepared according to the vendor’s protocol immediately before plating. Cells were placed on GFR-MG conditions as follows: 1) Par-C10 cells only (7000 cells/cm^2^), 2) m-CN cells only (50,000 cells/cm^2^), and 3) Par-C10 (7000 cells/cm^2^) and m-CN cells (50,000 cells/cm^2^) added simultaneously. Par-C10 cells plating density was chosen based on prior studies showing formation of polarized salivary epithelium when grown on GFR-MG^[13]^. For m-CN, we chose the plating density from a range of values reported in previous studies^[17–19]^. Plated cells were cultured for 4 days in a 37°C incubator with 95% air and 5% CO_2_, with medium replacement every 48 h as described previously^[13,14]^. The growth medium composition is detailed in (Table 1). Cell growth was monitored using phase contrast imaging with an EVOS XL Core light microscope (Thermo Fisher, Waltham, MA).

### Staining and imaging

Cells on GFR-MG as well as frozen sections from mouse SMG were fixed in 2% paraformaldehyde for 10 min at room temperature, incubated with 0.1% Triton X-100 in phosphate buffered saline (PBS) for 5 min followed by incubation with 5% goat serum for 1 h at room temperature. Antibodies were all diluted in 5% goat serum prior to incubation. Cells were incubated overnight at 4°C with rabbit anti-ZO-1 (1:200 dilution; Invitrogen, Carlsbad, CA) and mouse anti-β-3-tubulin (1:500; Abcam, Cambridge, MA), then washed three times with PBS, incubated for 1 h with AlexaFluor 488-conjugated goat anti-rabbit and AlexaFluor 568-conjugated goat anti-mouse (both 1:500 in 5% goat serum; Sigma, St. Louis, MO), washed three times with PBS, and stained for 10 min with TO-PRO nuclear stain (1:5000 dilution in PBS). Immunofluorescence images were obtained using a Zeiss LSM 700 confocal laser-scanning microscope (Carl Zeiss Microscopy, Thornwood, NY). Cell cluster diameter was measured using ImageJ software (National Institutes of Health, Bethesda, Maryland).

### Statistics

Unpaired two-tailed t-tests were used to determine whether there is a significant difference in: 1) size of Par-C10 cell clusters in single cultures vs. co-cultures, and 2) size of Par-C10 cell clusters with m-CN vs. no m-CN in co-cultures. Data shown are means ± S.E.M. of results from three experiments, where *P* values < 0.05 were considered significant.

## Results and Discussion

**Figure 1:**
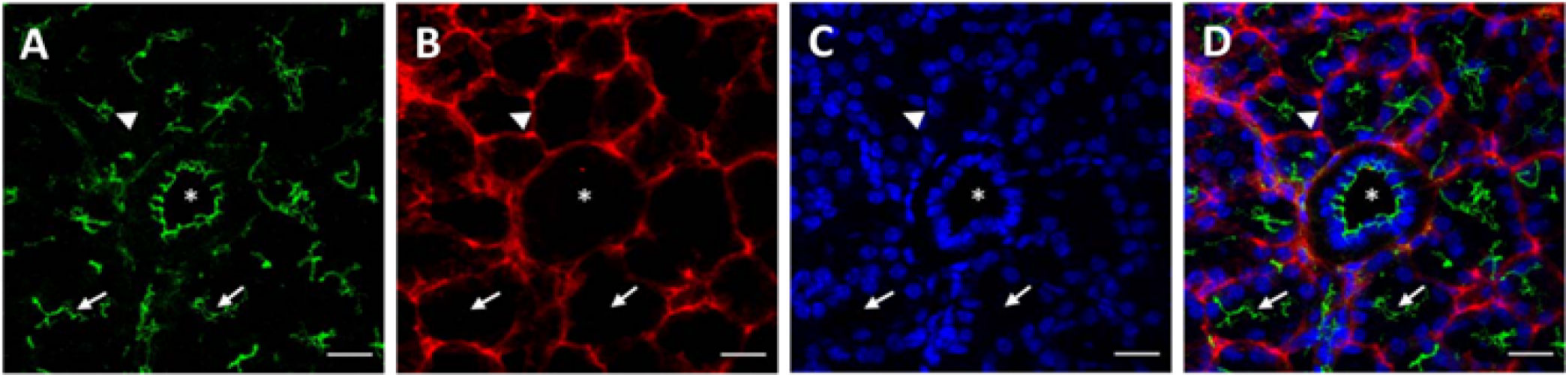
Native salivary gland tissue exhibits neuronal organization around salivary epithelial structures Immunofluorescent images taken at 20× magnification reveal a distribution of neurons on the basolateral side of salivary epithelial structures, including acini (triangles), striated ducts (arrows), and excretory ducts (asterisks). Scale bars are 20 µm. (A) ZO-1 (green) is observed lining luminal spaces in acinar and ductal structures. (B) B-3-tubulin staining (red) indicative of neuronal cytoskeleton is distributed basolateral to epithelial structures. (C) TO-PRO (blue) staining indicates cell nuclei. (D) Overlay image.

**Figure 2:**
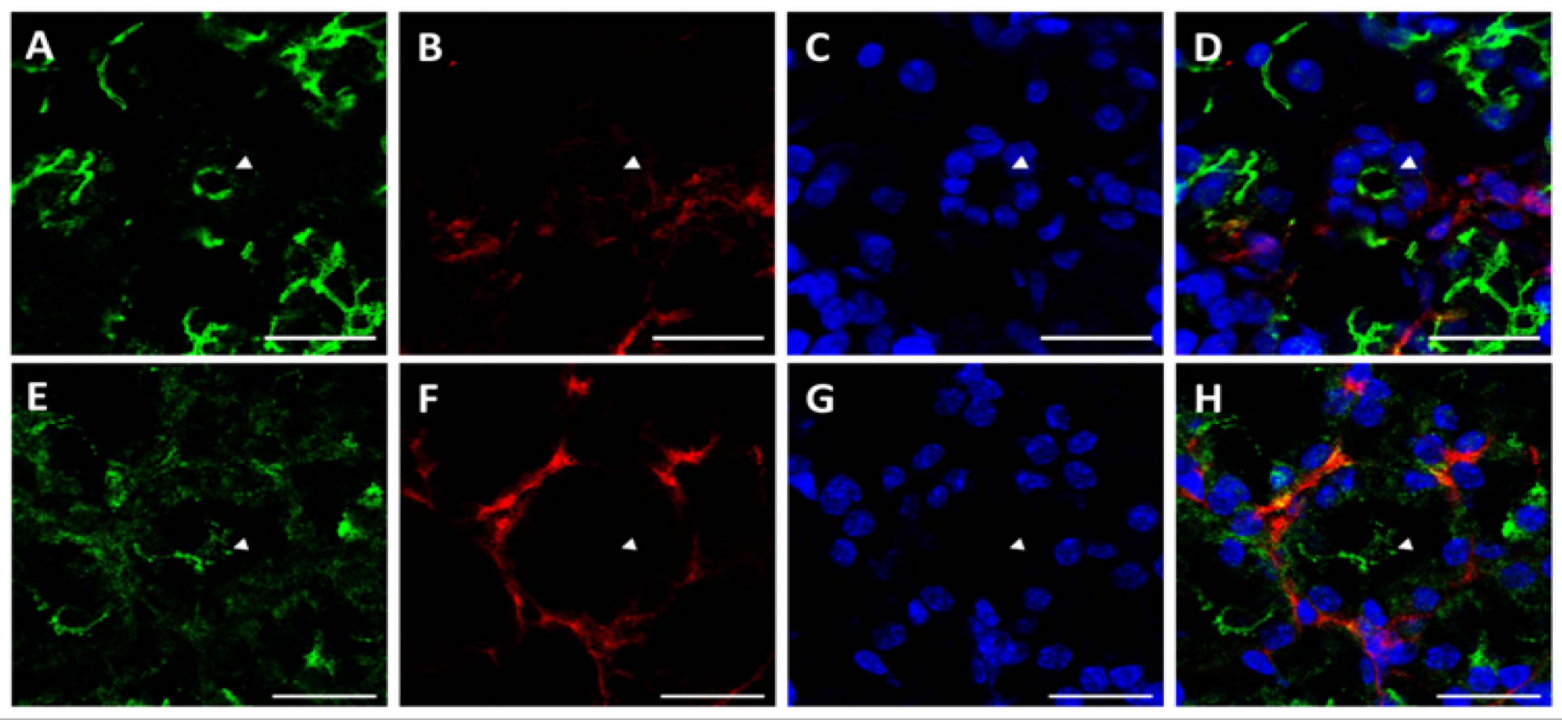
Neuronal distribution around acini in native salivary gland tissue is non-uniform Immunofluorescent images taken at 63× magnification reveal a highly non-uniform distribution of neurons on the basolateral side of acini (arrow-heads indicate lumens). Scale bars are 20 µm. (A&E) ZO-1 (green) marks the apical surface of acini. (B&F) B-3-tubulin staining (red) is distributed in a non-uniform manner around the basolateral surface of acini. (C&G) TO-PRO (blue) staining indicates cell nuclei. (D&H) Overlay images.

**Figure 3:**
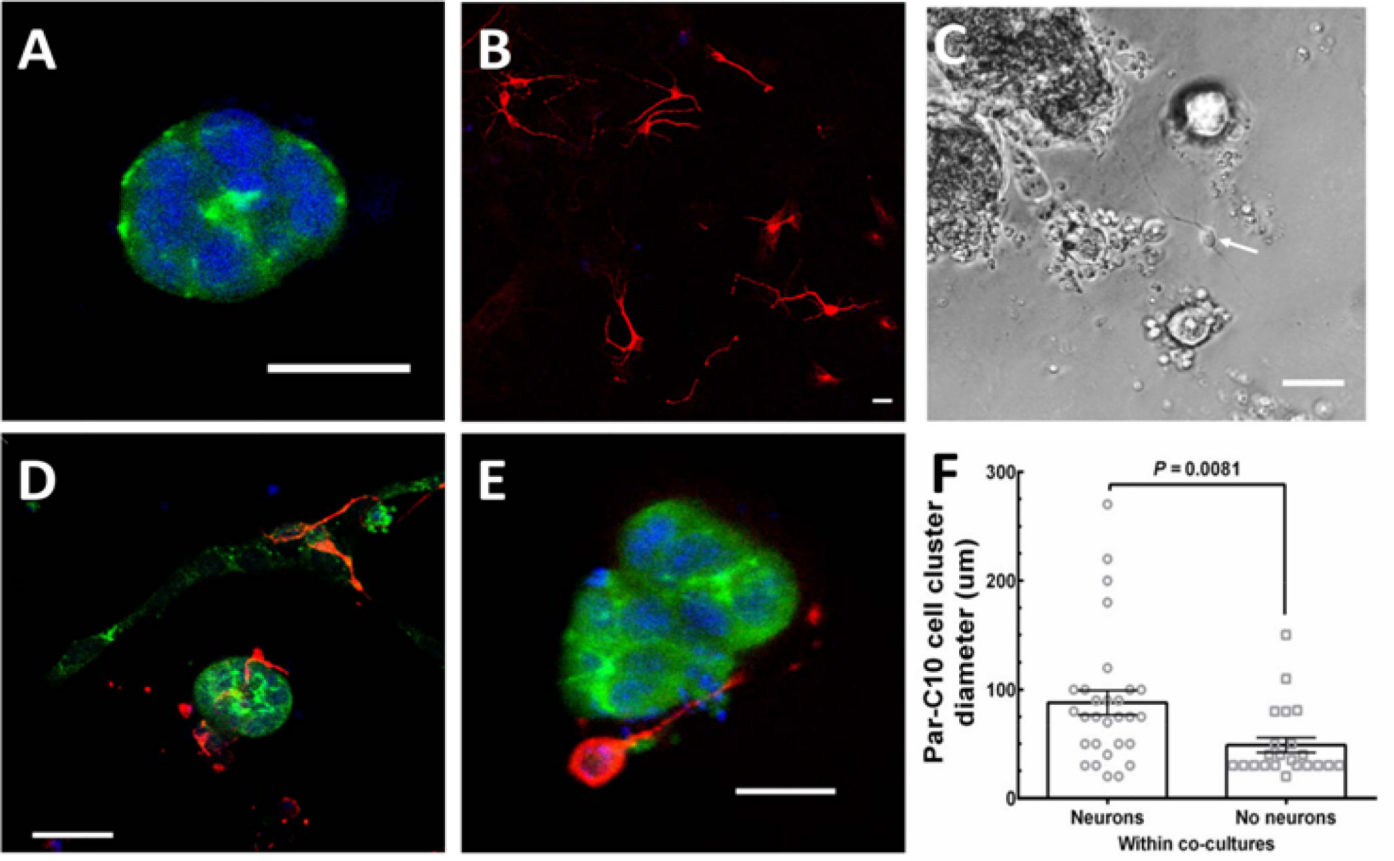
m-CN cells self-organize around Par-C10 cell clusters (A) Par-C10 cells grown on GFR-MG form salivary cell clusters with apical ZO-1 (green) staining. (B) m-CN cells, exhibiting β-3-tubulin (red) staining, grown on GFR-MG form cell monolayers. (C) Phase contrast image showing Par-C10 cells forming polarized salivary epithelial structures of various sizes with hollow lumens, and individual cells on the basolateral side. A cell with visible processes, indicative of an m-CN, can be observed (arrow). (D) Par-C10 and m-CN co-cultures form organized salivary cell clusters surrounded by basolateral m-CN (three-dimensional view). (E) A cross-section of a Par-C10 cell cluster surrounded by basolateral m-CN in co-cultures. (E) For Par-C10 clusters in co-cultures with m-CN, the diameter of Par-C10 cluster with neurons (88.1 ± 11.2 µm) was significantly larger than Par-C10 without neurons (48.91 ± 6.86 µm, P = 0.0081). Bars represent mean ± S.E.M. Scale bars for (A), (B), (D), (E) are 20 µm. Scale Bar for (C) is 100 µm.

Sections from mouse submandibular glands displayed typical salivary structures in tissue sections stained with H&E, including acini, striated ducts, and excretory ducts [Supplementary Figure 1]. Using structures identified by H&E staining as a reference, we were able to identify the same salivary structures in immunofluorescence images. As shown in (Figure 1A), we observed apical ZO-1 (green) lining luminal spaces in acini (triangles), striated ducts (arrows), and excretory duct (asterisk), indicative of healthy salivary gland tissue. ZO-1 is part of a multiprotein complex present on the apical surface of both acinar and ductal structures in all major salivary glands, and is important for proper saliva secretion^[20,21]^. We also observed non-uniform expression of β-3-tubulin (red) around acinar and ductal structures, exhibiting higher intensity nodes with lower intensity projections towards the basolateral epithelial surfaces, as shown in (Figure 1B and Figure 1C) shows cell nuclei, while (Figure 1D) shows an overlay image. The non-uniformity of β-3-tubulin can be better visualized around acinar structures at 63×, where some acini, identified by apical ZO-1 staining (Figure 2A and 2E), will only show diminished neuron staining on one direction the basolateral side (Figure 2B and 2F). (Figure 2C and 2G) show cell nuclei, while (Figure 3D and 3H) show an overlay image. β-3-tubulin is a microtubule protein from the β-tubulin family expressed exclusively in central and peripheral neurons^[22]^, and has been used as a neuronal marker in a variety of *in vitro* and *in vivo* studies^[23,24]^. The observed pattern of β-3-tubulin staining indicates a branching neuronal morphology typical of the salivary gland^[25]^, which can explain the non-uniformity of β-3-tubulin staining as innervation of salivary structures by progressively fewer numbers of neurons as the salivary structures get smaller. Our findings are the first report on overall neuronal distribution using confocal microscopy within the adult salivary gland for the purpose of developing an *in vitro* model.

As shown in (Figure 3A) Par-C10 cell clusters grown on GFR-MG formed polarized three-dimensional structures with apical ZO-1 (green) staining. Conversely, m-CN grown on GFR-MG only formed monolayers (Figure 3B), expressing β-3-tubulin (red) without ZO-1 staining. (Figure 3C) is a phase-contrast image showing an example of a co-culture, with Par-C10 cells forming spherical clusters of various sizes with hollow lumens similar to the organizational pattern that occurs in native salivary gland tissue. Individual cells can be observed on the basolateral surface of these Par-C10 cell clusters, and a cell with extended processes suggestive of a neuron is indicated with a white arrow. When both cell types were plated simultaneously on GFR-MG, we observed that m-CN monolayer formation was reduced, and m-CN instead grew three-dimensionally to surround the basolateral surfaces of Par-C10 cell clusters (Figure 3D and 3E), ZO-1: green, β-3-tubulin: red). This self-organization of m-CN was observed in 60% of Par-C10 cell clusters in co-cultures, and we did not observe any m-CN within Par-C10 cell clusters, only on the basolateral surface. No significant size difference was observed between Par-C10 cell clusters in single cultures vs co-cultures, indicating that m-CN did not interfere with Par-C10 cell cluster formation. Finally, in neuron-salivary epithelial cell co-cultures, the diameter of Par-C10 cell clusters with basolateral m-CN was significantly larger (88.10 ± 11.20 µm) than Par-C10 cell clusters with no m-CN (48.91 ± 6.863 µm, P = 0.0081) (Figure 3F). This finding suggests that m-CN cells exhibit a preferential attachment to larger Par-C10 cell clusters

Before studying functional aspects of neuron-salivary epithelial cell connections, it is crucial to understand the structural organization between neurons and salivary epithelial cells in culture. A robust *in vitro* co-culture model can provide an excellent approach to isolate and study factors governing neuron-salivary epithelial cell interactions. Here, we describe self- organization of neurons around salivary epithelial cell in neuron-salivary epithelial co-cultures that is similar to native tissue. Our results indicate that m-CN are able to self-organize around Par-C10 cell clusters under growth conditions optimized for Par-C10 cell growth. Self-organization has been previously observed in different types of co-cultures^[26–28]^. Additionally, the co-culture of neurons with other cells types, such as endothelial and muscle cells, have been shown to be achievable^[7,8,29]^. In summary, our study is the first to both show the feasibility of co-culturing neurons with salivary epithelial cells and to demonstrate *in vitro* neuronal self-organization around salivary epithelial cells similar to native salivary gland tissue.

### Study Limitations

This study provides a proof of concept for a novel *in vitro* model to study neuron-salivary epithelial cell interactions. Many factors need to be optimized to produce a robust *in vitro* model capable of modeling functional connections between neurons and salivary epithelial cells. Specifically, culture conditions to optimize neuronal growth while maintaining Par-C10 cluster formation need to be further studied, including cell types utilized, plating densities, culture duration, and growth medium supplements. In this study, we used Par-C10 cells due to their ability to form polarized salivary structures. In addition, m-CN was chosen due to their commercial availability and ubiquity in the literature as a pan-neuronal cell model. Since the primary goal of this study was to describe the interactions between neurons and salivary epithelial cells *in vitro*, we investigated the effect of m-CN on Par-C10 cell cluster formation, but not the inverse effect of Par-C10 cells on m-CN growth and morphology. It is certainly possible that the co-culture of Par-C10 with m-CN could have unintended effect on m-CN growth, and future studies will be conducted to investigate this possibility. Additionally, the Par-C10 immortalized cell line is derived from rat parotid glands, presenting a species compatibility issue for co-culture with m-CN. While the ultimate goal of generating a fully functional salivary gland *in vitro* would require the use of human salivary epithelial and peripheral parasympathetic and sympathetic neurons, the use of mixed co-cultures utilizing cells and cell lines from different species is well established for the development of *in vitro* models^[30,31]^. Our results provide a basis for an *in vitro* model that could allow future studies of complex cell-cell interactions between different cell types of the salivary gland.

## Supplementary Material

01

## Figures and Tables

**Table 1:** Composition of growth medium used for all culture conditions

Material	Vendor
Ham’s F12/DMEM (1:1)	Thermo Fisher
FBS (2.5%)	Thermo Fisher
Insulin-Transferrin-Selenite (10 µg/mL)	Sigma
Retinoic Acid (0.1 µM)	Sigma
EGF (80 ng/mL)	Sigma
T3 (3,3′,5 Triiodothyronine)	Sigma
Hydrocortisone (1.1 µM)	Sigma
Glutamine (5 mM)	Thermo Fisher
Gentamicin (50 µg/ml)	Sigma
