# Supplementary material for: Neurons Self-Organize Around Salivary Epithelial Cells in Novel Co-Culture Model
Source: J Stem Cell Regen Biol. Author manuscript; Available in PMC 2016 Nov 8. (PMC5100677; doi:10.15436/2471-0598.16.013)
Supplement: 01 [file NIHMS797168-supplement-01.pdf]

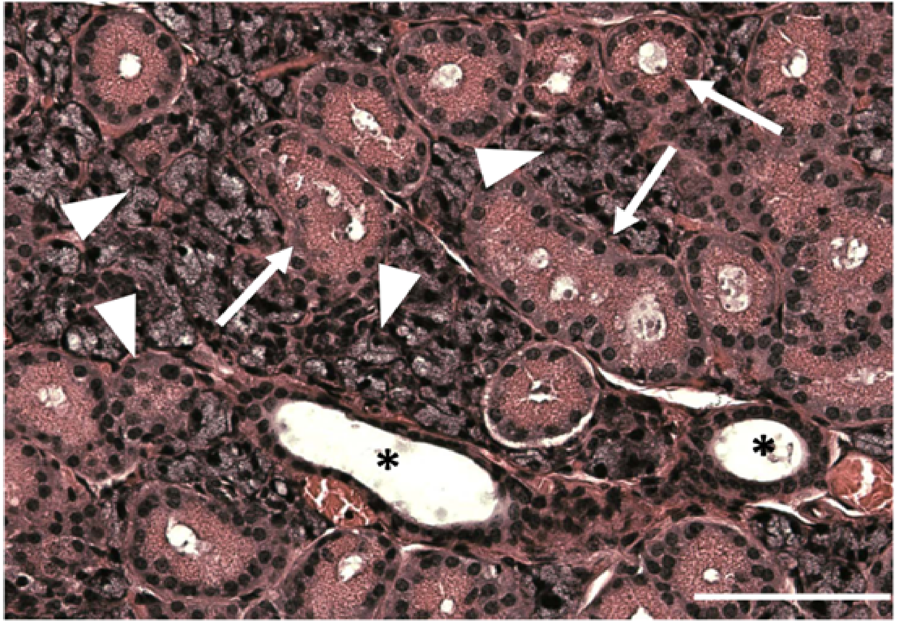

Supplementary Figure 1: Native salivary gland tissue displays acinar and ductal organization

H&E staining of mouse SMG reveals organized salivary epithelial structures, including acini (triangles), striated ducts (arrows), and excretory ducts (asterisks). Scale bar is 100 µm
